# Are SARS Superspreaders Cloud Adults?

**DOI:** 10.3201/eid1104.040639

**Published:** 2005-04

**Authors:** Stefano Bassetti, Werner E. Bischoff, Robert J. Sherertz

**Affiliations:** * University Hospital Basel, Basel, Switzerland;; † Wake Forest University School of Medicine, Winston-Salem, North Carolina, USA

**Keywords:** SARS virus, *Staphylococcus aureus*, transmission, letter

**To the Editor:** The primary mode of transmission of severe acute respiratory syndrome (SARS) appears to be through exposure to respiratory droplets and direct contact with patients and their contaminated environment. However, in summarizing their experiences during the SARS outbreaks in Toronto and Taiwan, McDonald et al. (*1*) note that certain persons were very efficient at transmitting SARS coronavirus (SARS-CoV), and that in certain settings these so-called "superspreaders" played a crucial role in the epidemic. Airborne transmission by aerosols may have occurred in many of these cases. The same observation has been made by others (*2**–**4*), but the causes of these superspreading events and the reasons for the variable communicability of SARS-CoV are still unclear. Possible explanations include specific host characteristics (e.g., altered immune status, underlying diseases), higher level of virus shedding, or environmental factors (*1**–**3*).

We hypothesize that superspreading events might be caused by coinfection with other respiratory viruses. Such a mechanism has been identified in the transmission of *Staphylococcus aureus*. Eichenwald et al. (*5*) showed that newborns whose noses are colonized with this bacterium disperse considerable amounts of airborne *S. aureus* and become highly contagious (i.e., superspreaders) after infection with a respiratory virus (e.g., adenovirus or echovirus). These babies caused explosive *S. aureus* outbreaks in nurseries. Because they are literally surrounded by clouds of bacteria, they were called "cloud babies" (*5*). We have shown that the same mechanism also occurs in certain adult nasal carriers of *S. aureus* ("cloud adults") (*6**,**7*). Reports indicate that viral infections of the upper respiratory tract facilitate the transmission of other bacteria, including *Streptococcus pneumoniae*, *S. pyogenes*, *Haemophilus influenzae*, and *Neisseria meningitidis* (*8*). Moreover, superspreading events have also been reported in outbreaks of viral diseases such as Ebola hemorrhagic fever and rubella (*3*).

Some observations suggest that coinfection with other respiratory viruses might cause superspreading events with airborne transmission of SARS-CoV. First, other viral pathogens, including human metapneumovirus, have been detected together with SARS-CoV in some patients with SARS (*4*). Second, few patients with SARS are superspreaders, and upper respiratory symptoms such as rhinorrhea and sore throat are a relatively uncommon manifestation of SARS (with prevalences of 14% and 16%, respectively) (*4*). Thus, some patients with SARS and upper respiratory symptoms might be coinfected with other respiratory viruses and become superspreaders. Interestingly, the report on a SARS superspreading event in Hong Kong explicitly states that the superspreader had presented with a "runny nose" (in addition to fever, cough, and malaise) (*3*). Therefore, upper respiratory symptoms might be a marker for highly infectious SARS patients. Future investigations, based upon either existing specimens from the last outbreak or newly collected specimens from any future outbreak, should focus on whether an association exits between SARS superspreading events and coinfection with other respiratory viruses.
